# Paraneoplastic cerebellar degeneration from an isolated nodal clear cell carcinoma of suspected gynecologic origin: case report and literature review

**DOI:** 10.1007/s00432-025-06404-6

**Published:** 2026-01-27

**Authors:** Claire Rose Kissinger, Aashima Gupta, Mina Aiad, Zachary Rothkopf, Melissa Wilson

**Affiliations:** 1 Temple University, Philadelphia, USA; 2 Department of Hematology and Oncology, St. Luke’s University Health Network, Bethlehem, USA

**Keywords:** Paraneoplastic cerebellar degeneration, Paraneoplastic syndrome, Clear cell carcinoma, Anti-Yo antibody

## Abstract

Paraneoplastic cerebellar degeneration (PCD) is a rare neurologic syndrome that often presents before an underlying malignancy is diagnosed. Patients typically develop subacute cerebellar symptoms—such as vertigo, dysmetria, disconjugate gaze, and dysarthria—before any tumor is detected. Establishing the diagnosis involves ruling out metastatic disease, treatment toxicity, infection, and metabolic disturbances, and often hinges on finding antineuronal antibodies (most commonly anti-Yo, also called type-1 anti-Purkinje cell antibody or PCA-1) in blood or cerebrospinal fluid (CSF). Anti-Yo positivity is classically linked to breast or gynecologic cancers and carries a poor prognosis. We describe a 56-year-old woman with a remote history of parotid basal cell adenocarcinoma who developed gradually worsening cerebellar dysfunction over several months, despite negative initial imaging and routine laboratory tests. When more extensive antibody testing revealed high-titer anti-Yo antibodies in serum and CSF, our suspicion for a paraneoplastic process increased. Subsequent FDG-PET/CT identified FDG-avid pelvic lymph nodes, and surgical pathology confirmed metastatic clear cell carcinoma in two iliac nodes, although the uterus, ovaries, and fallopian tubes were entirely benign. The patient received plasma exchange, intravenous immunoglobulin (IVIG), high-dose steroids, and adjuvant carboplatin/paclitaxel. Although she experienced some improvement in speech and upper-limb coordination, she remains non-ambulatory with persistent cerebellar deficits, three years after initial presentation. This case illustrates how anti-Yo PCD can foreshadow an occult nodal gynecologic malignancy without an identifiable primary tumor, highlighting the need for an extensive workup—including advanced metabolic imaging and, sometimes, empiric surgical exploration—when initial evaluations are unrevealing. Early tumor localization and treatment of both the malignancy and autoimmune response remain crucial to optimizing neurologic outcomes.

## Introduction

Paraneoplastic cerebellar degeneration (PCD) is an immune-mediated disorder in which autoantibodies target cerebellar Purkinje cells in the context of malignancy. Most often, it presents in women with breast or gynecologic cancers and may precede tumor detection by weeks or even months (Cao et al. 1999). Clinically, PCD typically manifests as subacute vertigo, nystagmus, gait instability, and dysarthria. Because these symptoms are nonspecific, they can mimic more common conditions, and brain imaging may be normal early on, a prompt diagnosis of PCD can be challenging (Leypoldt and Wandinger 2014). Detecting antineuronal antibodies, particularly anti-Yo (PCA-1), can confirm the suspected PCD diagnosis and guide the workup to undercover an underlying malignancy. Treatment must address both the malignancy and the autoimmune process, frequently combining immunosuppression, plasma exchange, and chemotherapy or surgery (Frings et al. 2005). Although most patients tend to stabilize rather than improve despite intensive treatment, those who show meaningful recovery often received earlier intervention—emphasizing the critical role of timely recognition and treatment.

## Case presentation

**Fig. 1:**
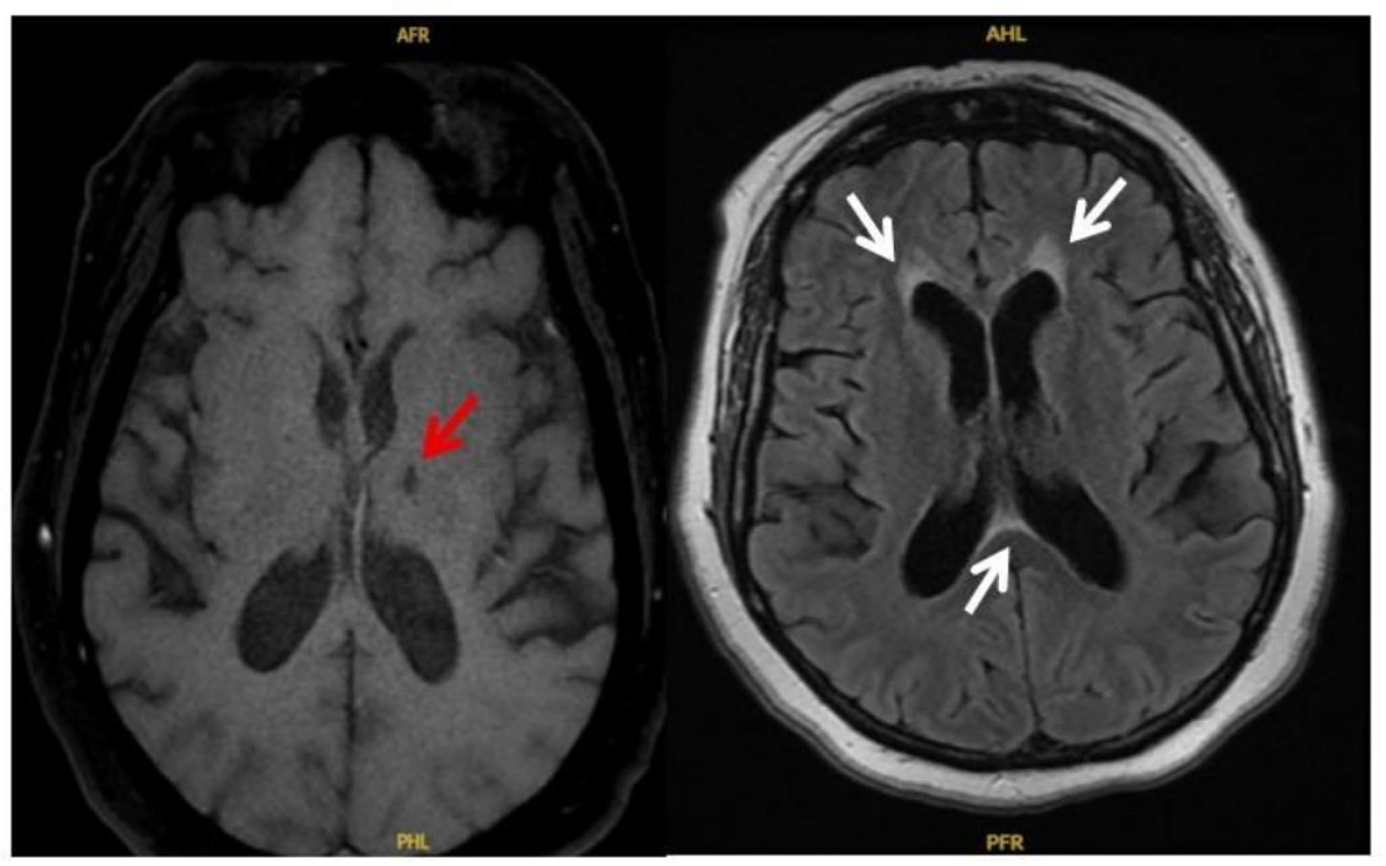
MRI Brain (Axial T2/FLAIR) demonstrating an old left thalamic lacunar infarct (red arrow) and small scattered periventricular white matter hyperintensities consistent with chronic microvascular changes (white arrows)

A 56-year-old woman with a history of pT4aN0 basal cell adenocarcinoma arising from a pleomorphic adenoma of the left parotid gland (treated 7 months prior to presentation with surgical resection and adjuvant chemoradiation) first reported acute-onset vertigo. Over the following few weeks, she presented multiple times to the emergency department. Her neurologic examinations remained non-focal, brain MRI showed only chronic microvascular changes and a stable old thalamic lacunar infarct (Fig. 1), and she was discharged with symptomatic measures (meclizine, and scopolamine).

One month after the time of presentation, she returned with persistent vertigo, nausea, and vomiting; evaluation revealed hypokalemia, which was corrected, and she was again discharged. However, later that same week, she developed bilateral lower extremity weakness and was admitted. Laboratory studies showed erythrocytosis (hemoglobin 18.3 g/dL, hematocrit 52.8% (reference: hemoglobin 12.0–16.0 g/dL, hematocrit 36–46% in females) and mild hyperbilirubinemia (total bilirubin 1.5 mg/dL (reference: 0.3–1.2 mg/dL); molecular testing for JAK2 V617F, CALR exon 9, and MPL exons 10–12 was negative. Otolaryngology noted left-sided hearing loss and nystagmus on Dix-Hallpike testing. CTA of the head and neck and repeat brain MRI ruled out local parotid adenocarcinoma recurrence. Hematology/oncology evaluation was unremarkable, and she underwent phlebotomy for presumed polycythemia. She was discharged with plans for outpatient neurology follow up and vestibular rehabilitation.

Three months after initial presentation, her cerebellar signs had progressed: she exhibited dysmetria, multidirectional nystagmus, and dysarthria. Concern for a paraneoplastic process prompted serum testing for autoimmune/paraneoplastic antibodies (Mayo Clinic MDS2 panel), which was initially negative. Given ongoing suspicion, an expanded paraneoplastic antibody panel (including CSF) was ordered. This revealed high-titer Purkinje cell cytoplasmic antibody type 1 (anti-Yo) in serum (1:3, 840) and CSF (1:64), strongly suggesting paraneoplastic cerebellar degeneration.

The patient subsequently received five sessions of plasma exchange (PLEX), five daily doses of IVIG at 400 mg/kg, and four days of high-dose IV methylprednisolone (1000 mg daily), with partial improvement in speech and upper-limb coordination.

To identify the occult malignancy, contrast-enhanced CT of the chest, abdomen, and pelvis five months after initial presentation revealed a 1.3 cm spiculated pulmonary nodule in the left upper lobe, a 1.3 cm left adrenal nodule, sclerotic lesions in the thoracolumbar spine, and endometrial thickening. Biopsies of the lung and adrenal lesions were benign. A nuclear medicine bone scan showed no osseous metastases. Endometrial curettage demonstrated benign inactive endometrium with focal non-atypical hyperplasia, without malignancy.

Seven months after initial presentation, the patient remained symptomatic with encephalopathy, dysarthria, nystagmus, and ataxia, but still no identifiable primary. An FDG-PET/CT scan then demonstrated FDG-avid lymphadenopathy along both iliac chains, enlarged compared to imaging two months earlier. Gynecologic oncology evaluation included pelvic washings, which revealed atypical cells positive for BerEp4 and pan-keratin (AE1/AE3) but negative for Napsin, P504S, WT-1, calretinin, and CK5/6 with background CD68-positive histiocytes—findings suspicious of malignancy.

Given these findings and her positive anti-Yo antibody status, surgical exploration was recommended. Because anti-Yo is strongly associated with gynecologic malignancy, and the location of the lymphadenopathy was not retroperitoneal or para-aortic, gynecologic oncology was favored over renal or urogenital.She underwent robotic total laparoscopic hysterectomy, bilateral salpingo-oophorectomy, and pelvic lymphadenectomy. Grossly, the uterus, cervix, and adnexa appeared normal. Pathology confirmed that uterine, ovarian, and fallopian tube tissues were benign. Of the pelvic lymph nodes, three left external iliac nodes were negative, but one right and one left common iliac node each harbored large metastatic deposits of clear cell carcinoma. Immunohistochemistry showed the tumor cells were positive for pancytokeratin (AE1/AE3), PAX8, CK7, P504S/Racemase, and Napsin A, and negative for CK20, p40, S100, inhibin, RCC marker, CD10, carbonic anhydrase IX, OCT3/4, and estrogen receptor; p53 expression was wild type. These findings, combined with anti-Yo positivity, pointed to a gynecologic origin despite no primary lesion in the uterus or ovaries.

Postoperatively, she received five days oral methylprednisolone course for neurological symptoms and then treated with adjuvant carboplatin (AUC 5) and paclitaxel (135 mg/m^2^), total of five cycles. Unfortunately, treatment was complicated by grade 4 neutropenia, pleural empyema, and pericardial effusion requiring drainage about ten months after initial presentation.

Approximately one year after initial presentation (approximately six months after surgery), she demonstrated modest improvements in upper-limb coordination and speech but remained non-ambulatory with bilateral lower extremity paralysis, persistent dysarthria, and mild cognitive impairment that limited decision-making.

At recent follow-up visits, three years after initial presentation, she remains alive without evidence of recurrent carcinoma but continues to require supportive care, physical therapy, and intermittent steroid use only if her neurologic symptoms worsen.

## Discussion

We reviewed 40 reported cases of anti-Yo-positive PCD, including our case (Tables 1, 2 and 3). The average age at diagnosis was 59.1 years (median 59.5; range 31–84), with a strong female predominance—37 patients (92.5%) were female and only 3 (7.5%) were male.

**Table 1:** Summary of paraneoplastic anti–Yo-positive PCD cases with primary malignancies identified

Reference	Age/ Sex	Cancer diagnosis (stage)	PCD onset before/after cancer diagnosis	Serum Anti-Yo	CSF Anti-Yo	Brain Imaging	Cancer treatment	PCD treatment	PCD symptoms at presentation	Duration of PCD symptoms before presentation	Outcome of PCD treatment & Survival	Survival after PCD diagnosis
Aparna et al. (2024)	68 F	High-grade serous ovarian carcinoma (clinically IIIC)	PCD ~ 4 months before cancer diagnosis	+	Not Reported (NR)	CT head: frontal & cerebellar atrophy in keeping with PNS, not age related	Debulking surgery + carboplatin/paclitaxel chemo	IVIG 1 g/kg with 1 g IV MP x total 5 doses	Progressive ataxia, dysarthria, falls, instability	~ 4 months	Slight improvement of speech and coordination, then cerebellar symptoms gradually worsened	Alive at 2 years
Baransi et al. (2022)	52 F	high-grade serous ovarian carcinoma; FIGO IIIB	PCD ~ 2 mo before cancer Dx	+	NR	Brain MRI: normal	Debulking surgery + adj Carbo/Taxol × 6, then niraparib 200 mg QD × 36 Mo	IV MP 1 g × 5 days, Cyclophosphamide 750 mg/m^2^ IV every 4–6 weeks	Gait ataxia, dysphagia, hand weakness & weight loss	~ 1 months	slight improvement in speech & swallowing, but her gait ataxia & limb incoordination persisted	≥ 3 years
Bhargava et al. (2014)	44 F	Borderline serous papillary ovarian carcinoma (FIGO IIA)	PCD ~ 8 months before tumor found	+	Negative (-)	Brain MRI: Bilateral cerebellar atrophy	AHBSO	IV MP 1 g QD × 5 days, then IVIG course 2 g/kg over 5 days	Vertigo, nausea, vomiting; dysarthria, ataxia, nystagmus	8 months	Two weeks after AHBSO: marked improvement in dysarthria &vertigo By 1 month: ambulate with support By 2 Mo: only minimal residual ataxia & ocular findings; independent in ADLs	Alive &functionally independent at ~ 10 months
Bradley et al. (2008)	56 F	Serous ovarian carcinoma (III C)	PCD ~ 2.5 years after cancer Dx	+	NR	Brain MRI: normal	debulking surgery + Carbo/Taxol then more chemo for recurrence	PLEX, IVIG, corticosteroids, & Tacrolimus (dose: NR)	Difficulty walking, ataxia, dysarthria	~ 1 week	No neurological improvement	Alive at 24 months
Cao et al. (1999)	65 F	Poorly differentiated serous adenocarcinoma of the right ovary, FIGO IIIC	PCD ~ 1 month before cancer Dx	+	NR	Brain MRI: normal	Debulking surgery only	None – no PLEX, IVIG, or steroids	Dysarthria, gait ataxia, dysphagia, diplopia	3–4 weeks	No improvement remained in bedridden	Alive at 6 months
Ćerimagić and Džananović (2020)	61 F	HER2 positive invasive breast carcinoma, Stage IIA	PCD ~ 6 months before cancer diagnosis	+	NR	Initial MRI normal; follow-up at 3 months: 5 mm cerebellar lesion (ischemic vs. PCD	Neo chemo + HP, mastectomy, XRT & trastuzumab × 2 cycles	PLEX × 5, IV cyclophosphamide 1 g, azathioprine, IVIG 2 g/kg × 1 then 0.4 g/kx 13	Sudden onset vertigo, nystagmus & nausea	~ 10 months	Mild improvement in limb coordination after PLEX but remained wheelchair-bound with persistent ataxia & dysarthria	Alive at ~ 2–3 years
Chatham and Niravath (2021)—Case 1	57 F	HER2 positive invasive ductal carcinoma of breast, stage IIIA	PCD ~ 6 months before cancer Dx	+	+	Brain MRI: normal	Left mastectomy + trastuzumab plus pertuzumab × 17 cycles	IV MP + IVIG, PLEX × 2, & Rituximab over 7 Mo (dose: NR)	Progressive ataxia and dysarthria	~ 3 months	No significant improvement – PCD stabilized with severe deficits (wheelchair-bound, dysarthria persists)	Alive at 39 months
Chatham and Niravath (2021)—Case 2	61 F	HER2 positive breast carcinoma	PCD ~ 3 months before cancer Dx	+	NR	Brain MRI: normal	Left mastectomy + trastuzumab plus pertuzumab × 17 cycles	PLEX over 7 Mo then Rituximab over 10 Mo (no doses, steroid or IVIG were reported)	Vertigo, nausea, vomiting & ataxia	~ 3 months	Minimal improvement – remains wheelchair-bound (able to take only a few assisted steps), with persistent dysarthria and poor coordination	Alive at 36 months
Cui et al. (2017)	65 F	Serous ovarian carcinoma (Stage III)	PCD 1 year after cancer diagnosis	+	NR	Brain MRI: normal	Cytoreductive surgery + adj cisplatin/paclitaxel × 7	IVIG 1 g × 7 days + IV MP 0.4 g/kg/day × 5 days	Rapidly progressive ataxia, vertigo & dysarthria	~ 5 months	Vertical nystagmus resolved, speech improved, & regained ability to sit/stand (with supervision) & to mobilize in bed	≥ 19 months
Deac et al. (2021)	59 F	High grade serous ovarian carcinoma (Stage IV)	~ 4 months before cancer Dx	+	NR	Brain MRI: cerebellar atrophy, enlargement of 4th ventricle, scattered T2-signal abnormalities in the frontal lobes	paclitaxel–carboplatin x total of 6 cycles	IVIG 2 g/kg over 5 days + corticosteroid (dose: NR)	Vertigo, imbalance, bilateral plantar paresthesia, hedahche, insomnia, sad mood, & weight loss	~ 4 months	only a slight transient improvement of diplopia & vomiting—but cerebellar dysfunction progressed	~ 7 months
Elomrani et al. (2014)	80 F	Poorly differentiated ovarian adenocarcinoma	PCD and cancer diagnosed concurrently	+	NR	Brain MRI: normal	No resection; received Carboplatin + paclitaxel (dose NR)	Corticosteroids were given along with antiemetic with the chemo (Dose: NR)	subacute vertigo, vomiting, spontaneous nystagmus and clinical ataxia (on exam)	described as “subacute”	Complete disappearance of the cerebellar syndrome after the first chemo cycle; however, following the second cycle the ataxia recurred with marked deterioration in general condition	~ 1 months
Frings et al. (2005)– case 1	59 F	Adenocarcinoma of the left fallopian tube, at least FIGO Stage IIB	Several weeks	+	+	Initial brain MRI: normal Follow-up MRI: marked cerebellar atrophy, sparing the brain stem	Combined ovariectomy + adnexectomy, but no chemo was given	IV MP & Mitoxantrone (dose: NR)	Nystagmus, dysarthria, and severe ataxia	Weeks to months	No clinical improvement; remained severely ataxic and bedridden	NR
Frings et al. (2005)–case 2	57 F	Invasive lobular breast cancer, ER/PR negative, HER2 positive, ≥ IIIB	Few weeks	+	+	Brain MRI: advanced cerebellar atrophy without brain-stem involvement	No resection or XRT; received 5-fluorouracil + epirubicin + cyclophosphamide (FEC regimen)	anti-cancer chemo only	Nystagmus, dysarthria, and severe ataxia	Weeks to months	No clinical improvement	NR
Giucca et al. (2023)	~ 65 F	High-grade serous ovarian adenocarcinoma, FIGO stage IV	PCD ~ 16 months after cancer dx	+	NR	Brain MRI: normal	carboplatin + paclitaxel, interval debulking surgery, Bevacizumab added at cycle 6 then as maintenance	PLEX with IV MP 1 g/day × 5 &	double vision, dizziness, nausea, headache, and poor coordination	~ 9 days	Cerebellar syndrome continued to worsen despite treatment	~ 2 months
Hasadsri et al. (2013)	49 M	Large cell, poorly differentiated adenocarcinoma of the lung, Stage IA1 found at autopsy	~ 8 weeks between PCD onset and postmortem cancer diagnosis on autopsy	NR	+	Brain MRI: mild generalized cerebral and cerebellar atrophy	*No oncologic therapy* (cancer found post-mortem)	IV MP (dose: NR)	Vertigo, gait ataxia, slurred speech; falls and vomiting	~ 2 weeks	Initial improvement but later worsened, progressive ataxia, became bedridden; fatal outcome	Hasadsri et al. (2013)
Imai et al. (2022)	60 F	Left submandibular gland salivary duct carcinoma	PCD onset led to workup revealing the cancer (5 days apart)	+	NR	Brain MRI: normal	Tumor resection (submandibular glandectomy + neck dissection); no adjuvant therapy noted	IV MP 1 g/d × 3, PLEX × 10, IVIG × 5 days, cyclophosphamide (dose: NR)	Acute gait ataxia, limb and trunk dysmetria, truncal instability	~ 5 days	Partial improvement with Nausea, vertigo and nystagmus after tumor resection but persistent ataxia	Not Reported (alive at report, persistent PCD)
İlhan et al. (2021)	54 F	Ovarian cancer, recurrent after initial remission	PCD ~ 3 years after cancer remission, and ~ 6 month before recurrence dx	+	+	Brain MRI: normal	Cytoreductive surgery at initial diagnosis; after recurrence, received ed carboplatin + paclitaxel × 6 cycles	Tingling & unsteadiness while walking, ataxia, dysarthria	PLEX × 5, each performed 2 days prior to the chemo	~ 6 months	Significant improvement in walking and balance noted by the start of cycle 2 of chemotherapy	NR
Kato et al. (2017)	42 F	Left breast microinvasive ductal carcinoma, ER/PR negative, HER2 positive, stage IA	PCD ~ 2 months before cancer dx	+	+	Initial Brain MRI: normal; 4-months post-surgery: cerebellar atrophy	Left mastectomy (no adjuvant therapy)	IVIG, steroids, and PLEX (does/ number of treatments: NR)	Dizziness, dysarthria, ataxia, slightly impaired orientation	~ 2 months	Initially improved, then relapsed – residual ataxia despite immunotherapy	≥ 1 year
Le May and Dent (2018)	53 F	invasive ductal carcinoma of the left breast, ER/PR negative, HER2 positive, stage IIB	PCD ~ 4 months before cancer dx	+	NR	Brain MRI: normal	Mastectomy, adjuvant Doxorubicin–cyclophosphamide × 4, Paclitaxel weekly × 12 cycles with trastuzumab	IVIG × 2 doses & Physiotherapy	ataxia, dysarthria, emotional lability, diplopia & fine motor weakness	~ 4 months	No meaningful improvement after IVIG or chemotherapy; PCD deficits stabilized but persisted, with eventual wheelchair dependence and long-term care placement	≥ 9 months
Liapi and Sarivalasis (2020)	61 F	High-grade serous ovarian cancer (FIGO IIIC)	PCD at presentation – preceded cancer diagnosis	+	NR	Brain MRI: diffuse cerebellar atrophy, predominant in vermis	carboplatin/ paclitaxel & maintenance Bevacizumab × 15 month	Anti-cancer chemo	gait ataxia, dysphonia, dysarthria, dysmetria	NR	Transient improvement of ataxia during chemo	≥ 7 years
Lie et al. (2016)	84 F	High-grade serous endometrial adenocarcinoma (recurrence with LN metastasis)	PCD onset ~ 1 year after initial cancer, and ~ 6 months before the recurrence dx	+	NR	Brain MRI: normal	AHBSO at initial cancer diagnosis	High dose steroid (dose: NR)	gait ataxia, dysarthria, tremors, nystagmus	6 months history of falls	NO improvement; severe functional decline	NR
Martin et al. (2017)	52 F	Breast infiltrating ductal carcinoma (stage IIIA, HER2 positive)	Breast cancer was uncovered on chest CT performed at her initial PCD presentation	+	NR	Brain MRI: chronic microvascular changes	Neo docetaxel + carboplatin + HP, mastectomy, adj XRT, adj HP. Later lapatinib + capecitabine; ado-trastuzumab (for recurrence)	IVIG 2 g/kg total, given over 3 days, then maintenance monthly	Subacute ataxia, dysarthria, diplopia, dysphagia, nystagmus; episodes of uncontrolled crying (pseudobulbar affect)	~ 1 month	Transient—but significant improvement in ataxia and pseudobulbar affect, slight dysarthria improvement, but later, she retained severe truncal and limb ataxia	≥ 3 years
Mirallas et al. (2020)	44 F	Multifocal invasive breast ductal carcinoma, HER2 positive, stage IA	PCD ~ 5 months after breast cancer dx	+	+	Brain MRI: normal	Neo Doxorubicin + Cyclophosphamide × 4 – paclitaxel/HP × 11, mastectomy, adj trastuzumab/tamoxifen	IVIG course over 5 days	Gait instability, right upper extremity weakness, mild paresthesia × 4 extremities, discrete right diadochokinesia	~ 5 weeks	significant improvement, autonomous gait restored, able to sit/stand unaided but had persistent right-sided asymmetry and mild balance disorder	≥ 1 year
Othman et al. (2020)	48 F	Serous carcinoma arising from fallopian tube/peritoneum	~ 2 weeks before cancer dx	+	NR	Brain MRI: normal	1 cycle carboplatin alone; then 5 cycles carboplatin + paclitaxel	Anti-cancer chemo	Falls, ataxia, dysarthria, hyperreflexia, limb dysmetria	~ 2 weeks	Mild improvement: at 18 months follow-up able to move extremities and transfer with assistance	≥ 18 months
Renjen et al. (2018)	65 F	High-grade serous ovarian carcinoma (FIGO IIIA1)	PCD ~ 2 months before cancer dx	+	NR	Brain MRI: diffuse cerebellar folial enhancement &mild atrophy	Debulking surgery + adj carboplatin/paclitaxel	IVIG × 5 days	Subacute‐onset imbalance/ataxia & sudden throbbing headaches	~ 2 months	Partially regained ambulation but remained ataxic	≥ 6 months
Russo et al. (2013)	64 F	High grade serous papillary ovarian cancer (FIGO IIIC)	PCD 1 year after cancer diagnosis	+	+	Brain MRI: normal	Reduction surgery + adj Carboplatin/Paclitaxel × 6 cycles	IVIG 400 mg/kg daily × 7 + corticosteroids + Cyclophosphamide 800 mg/m^2^/month	Subacute dysmetria, ataxia, dysgraphia, nystagmus, diplopia & mild dysphagia	~ 2 months	Stabilization only; no significant improvement; remained severely disabled	≥ 12 months
Tanaka et al. (1992)	47 F	Fallopian tube adenocarcinoma, FIGO IA	PCD ~ 3 months before cancer dx	+	+	Brain MRI: small old cerebellar infarct, otherwise unremarkable	Tumor resection via test laparotomy + adj FCAP (cisplatin, Adriamycin, cyclophosphamide, 5-FU) × 3 cycles	Anti-cancer treatment only	Acute diplopia, ataxia, vertigo, nausea, vomiting, ataxia, & upper limbs hyperreflexia	~ 3 months subacute	regained ability to sit unaided, improved speech; autoantibody titers fell	≥ 1 year
Wang et al. (2022)	51 F	Invasive ductal breast adenocarcinoma, ER/PR negative, HER2 positive, stage IIIC	PCD ~ 33 days before cancer dx	+	+	Brain MRI: normal	Mastectomy + adj paclitaxel, carboplatin, HP × 2 cycles only	IVIG 5 days (40 g Day 1, 25 g × 4) + high-dose IV MP then taper to oral prednisone	Unsteady gait, dizziness, dysarthria (explosive speech), vertigo; rapidly progressive cerebellar ataxia with pyramidal signs	~ 6 weeks	Neurologic decline halted shortly after immunotherapy; at 1-year follow-up: no cancer recurrence, neurologic symptoms stable (persistent ataxia/dysarthria without further deterioration)	≥ 1 year
Westerlinck et al. (2022)	31 F	Invasive ductal breast carcinoma, triple positive, clinical stage IIB	PCD ~ 1.5 months before cancer dx	+	NR	Brain MRI: initially normal; repeat showed bilateral cerebellar T2 FLAIR hyperintensity without contrast enhancement	Neo EC (epirubicin/ cyclophosphamide), mastectomy, adj EC × 2, then paclitaxel + HP × 13 cycles + XRT	IVIG 400 mg/kg/day × 5, High-dose corticosteroids, Natalizumab monthly × 6 & Whole-brain RT	headache, dizziness, ataxia, dysmetria, nystagmus, diplopia, dysarthria / expressive aphasia	~ 1 month	Rapid stabilization after whole-brain RT + tumor resection; over months: clear speech, improved motor skills & continued neurological recovery	≥ 33 months
Xia et al. (2003)	55 M	Invasive adenocarcinoma of distal esophagus, stage IIIB	PCD ~ 6 weeks before cancer dx	+	+	Brain MRI: large cisterna magna noted, otherwise unremarkable	Cisplatin + Irinotecan, palliative XRT, then esophagectomy	Empiric immunosuppressants (unspecified), IVIG and PLEX (unspecified)	Progressive ataxia, dizziness, dysarthria, nystagmus, dysmetria of all extremities & weight loss	~ 6 weeks	No neurologic improvement; became wheelchair-bound & mute postoperatively	≥ 6 months
Yan et al. (2018)	67 F	Invasive ductal breast carcinoma, ER/PR negative, HER2 positive, stage IIA	PCD ~ 6 months before cancer dx	+	NR	Brain MRI: normal	Left breast lumpectomy; no adj therapy	IVIG (unspecified)	Dizziness, dysarthria, nausea, vomiting, diplopia& psych symptoms	~ 6 months	Marked neurologic improvement after lumpectomy; at 12-month follow-up: resolution of ataxia, dysarthria, and gait instability	≥ 12 months
Yang et al. (2025)	62 F	Squamous cell lung carcinoma; stage IIIB	PCD ~ 3 weeks before cancer dx	+	NR	Brain MRI: normal	Cisplatin/etoposide × 3 + Concurrent thoracic radiotherapy	IVIG 0.4 g/kg/d × 5 + IV MP 80 mg/day × 7 then po prednisone taper + Mycophenolate mofetil	Vertigo, nausea, vomiting, ataxia, nystagmus and diplopia	~ 3 weeks	Significant improvement of nausea, vomiting & vertigo; gained independent ambulation without gait support	≥ 6 months
Yasuda et al. (2024)	77 F	Peritoneal serous papillary carcinoma, FIGO IIB	PCD ~ 5 months before cancer dx	+	NR	Bilateral cerebellar atrophy on MRI (with cerebellar hypoperfusion on SPECT)	THBSO + adj paclitaxel/ carboplatin × 6 cycles	Monthly IVIG (0.4 g/kg/day × 5 days) concurrent with chemotherapy (6 courses)	subacute dysarthria with scanning speech, Neck tremor, severe limb dysmetria & ataxia	~ 5 months	Dysarthria began improving after 3rd IVIG course but had Persistent limb ataxia & wheelchair dependence	≥ 6 months
Zhuang et al. (2024)	57 F	Remote history of breast cancer ER/PR negative HER2 positive s/p resection	PCD occurred later, after cancer treatment (period: NR)	+	+	Brain MRI: leptomeningeal enhancement prominently involving both cerebellar hemispheres	No active oncologic therapy reported (no recurrence or metastasis identified)	PLEX + IV MP (dose: NR)	Gait unsteadiness, receptive aphasia, insomnia, cerebellar ataxia with nystagmus and diplopia	~ 12 days	No significant neurologic improvement	Patient died during follow-up (exact period: NR)

*ADLs* activities of daily living, *AH-BSO* Abdominal hysterectomy with bilateral salpingo-oophorectomy, *Adj* adjuvant, *BID* twice daily, *Dx* diagnosis, *F* female, *HP* trastuzumab-pertuzumab, *IV* intravenous, *IVIG* intravenous immunoglobulin, *LN* lymph node, *M* male, *MP* methylprednisolone, *Mo* months, *NR* not reported, *Neo* neoadjuvant, *PCD* paraneoplastic cerebellar degeneration, *PNS* paraneoplastic neurological syndrome, *PO* oral, *QD* once daily, *SPECT* Single-Photon Emission Computed Tomography, *XRT* radiation therapy

**Table 2:** Summary of paraneoplastic anti–Yo-positive PCD cases with no malignancy identified / occult malignancy

References	Age/ Sex	Presumed occult malignancy (no tumor identified)	Serum Anti-Yo	CSF Anti-Yo	Brain Imaging	Cancer treatment	PCD treatment	PCD symptoms at presentation	Duration of PCD symptoms before presentation	Outcome of PCD treatment & Survival	Survival after PCD diagnosis
Hu et al. (2020)	64 M	Workup included Whole-body PET/CT, CT chest/abdomen/pelvis, targeted endoscopic biopsies (e.g., ileal ulcer) all negative	+	−	MRI: normal	None	Dexamethasone 10 mg QD × 10 days; Plasma exchange (4 courses); Prednisone 10 mg QD × 3 months	Progressive ataxia, gait instability, dysuria, blurred vision, diplopia, blepharoptosis	~ 5 days	Marked improvement: independent ambulation; anti-Yo became undetectable; symptoms relieved at 6 months	≥ 6 months
Dou et al. (2024)	63 F	An ultrasound examination revealed lymphadenopathy and left side breast nodules, but PET/CT did not identify any tumors	+	−	MRI: initially normal; 5 months later cerebellar atrophy	Not applicable (no tumor identified)	IVIG 0.4 g/kg × 5 days; IV methylprednisolone 500 mg × 10 days; Ofatumumab 20 mg × 2 injections	Dizziness, vomiting, nystagmus, dysarthria, progressive ataxia	~ 1 month	Partial improvement: walker-dependent but able to stand; further symptomatic relief after second OFA dose	≥ 11 months

**Table 3:** Summary of paraneoplastic anti–Yo-positive PCD cases with isolated lymphadenopathy/ lymph node metastases

References	Age/ Sex	Cancer diagnosis (stage)	PCD onset before/after cancer diagnosis	Serum Anti-Yo	CSF Anti-Yo	Brain Imaging	Cancer treatment	PCD treatment	PCD symptoms at presentation	Duration of PCD symptoms before presentation	Outcome of PCD treatment & Survival	Survival after PCD diagnosis
Abdulaziz et al. (2018)	68 F	Biopsy of right axillary lymp nodes showed Poorly differentiated metastatic cancer cells but no primary found	PCD Dx led to finding LN cancer cells in LN	Positive ( +)	+	Brain MRI: normal	None	IV methylprednisolone (MP) 500 mg × 5 days, then PO prednisone 60 mg QD × 3 Mo	Vertigo, diplopia, nystagmus, severe ataxia	~ 6 months	Improved: walking independently at 1 year	Alive at 1 year
Chatham and Niravath (2021)—Case 3	79 F	Para aortic lymphadenopathy on image (3 cm),, presumed ovarian cancer Presumed metastatic ovarian cancer but patient declined biopsy	PCD ~ 4 months before cancer suspicion	+	NR	NR	Radiation therapy to the para-aortic/aortocaval LN	PLEX + Steroid, then IVIG (dose: NR)	Ataxia, blurry vision & fatigue	~ 4 months	Rapid neurologic deterioration – became completely unable to walk or speak by ~ 8 months	NR
Lou et al. (2021)	61 F	Biopsy of retroperitoneal lymphnodes showed adenocarcinoma of pancreaticobiliary origin, no primary found	PCD onset ~ 10 days before cancer dx	+	+	Brain MRI: normal	Anlotinib	IVIG 0.4 g/kg/day × 5, IV MP 160 mg/day × 3, then 80 mg/day × 3, then 40 mg/day × 7	vertigo, nausea/vomiting, nystagmus & ataxia	~ 10 days	Initial transient symptoms improvement then remained bedridden	≥ 16 months
Our case	56 F	Isolated clear cell carcinoma in two pelvic lymph nodes; presumed ovarian origin; primary not identified	PCD onset ~ 7 months before cancer dx	+	+	Brain MRI: chronic microvascular changes and stable thalamic lacunar infarct; no acute cerebellar lesion	Robotic hysterectomy, BSO, pelvic lymphadenectomy + adj carboplatin/paclitaxel × 5 cycles	PLEX × 5, IVIG 400 mg/kg/day × 5, IV MP 1000 mg/day × 4 days, then po prednisone taper	Vertigo, ataxia, bilateral lower extremity weakness, vertical diplopia, nystagmus, dysmetria & dysarthria	~ 3 months	modest recovery of coordination and speech after PLEX, IVIG, steroids; remained non‐ambulatory with bilateral lower extremity paralysis, dysarthria, and cognitive limitations	≥ 3 years

Among the 40 cases, the most frequent malignancy was ovarian cancer (15 cases, 37.5%): 11 serous ovarian carcinoma, 2 ovarian carcinoma unspecified type, 1 clinically presumed but no biopsy was done, and our case with clear cell carcinoma in inguinal lymph node presumed ovarian origin but no primary identified in the uterus, fallopian tubes and ovaries pathology.

Breast cancer was the second most common, reported in 12 cases (30%), all of them were HER2-positive. Other confirmed gynecologic cancers included fallopian tube serous carcinoma in 3 cases, serous endometrial carcinoma in 1 case, and primary peritoneal serous carcinoma in 1 case, all sharing high-grade serous histologic features.

Non-gynecologic malignancies were also represented: lung carcinoma (squamous and large cell, 2 cases—the large cell found at autopsy), esophageal adenocarcinoma (1 case), and cholangiocarcinoma (1 case, presumed based on lymph node biopsy). Additionally, 1 case involved submandibular gland carcinoma. One case found to have poorly differentiated cancer cells in axillary lymph node, but no primary identified. Two cases had no malignancy detected despite extensive evaluation.

Most patients—33 out of 40—presented with subacute PCD symptoms, evolving over weeks to months, while only 6 patients had an acute onset with symptoms developing over a few days.

In 30 cases, PCD was diagnosed before the underlying malignancy, with reported intervals ranging from a few days to 8 months, and an average of approximately 5 months. Notably, only seven cases were diagnosed with cancer within the same week as PCD. This highlights the potential for a significant delay between neurologic and oncologic diagnoses, underscoring the importance of prolonged and repeated malignancy surveillance in patients with paraneoplastic cerebellar degeneration.

On the other hand, although less common, 8 cases had PCD onset after the cancer diagnosis, with reported intervals ranging from 5 months to 3 years. In several instances, PCD developed during remission or in the setting of recurrence—notably, in two patients who manifested PCD approximately 6 months before confirmed cancer recurrence, despite having completed initial treatment 1 to 3 years earlier (İlhan et al. 2021; Lie et al. 2016). These observations demonstrate that PCD can emerge well after an initial cancer diagnosis or period of remission, reinforcing the importance of continued neurologic surveillance in oncology patients, especially those at risk for recurrence.

Brain MRI findings were reported in all but one of the 40 patients with anti-Yo–associated PCD. Twenty-four patients (60%) had normal or unremarkable brain MRI at the time of presentation, reflecting the often non-specific or delayed imaging findings in early PCD. Eight patients (20%) demonstrated cerebellar atrophy on initial MRI, and an additional three patients initially had normal imaging but developed cerebellar atrophy on repeat MRI, highlighting the progressive nature of cerebellar degeneration (Frings et al. 2005; Kato et al. 2017; Dou et al. 2024). Isolated cases revealed other abnormalities: one patient had a focal cerebellar lesion (Ćerimagić and Džananović 2020), another had a cerebellar infarction (Tanaka et al. 1992), and one patient showed bilateral cerebellar T2 FLAIR hyperintensities without contrast enhancement (Westerlinck et al. 2022). Another had leptomeningeal enhancement prominently involving both cerebellar hemispheres (Zhuang et al. 2024). These findings show that while early brain MRI is often normal in PCD, serial imaging can reveal evolving cerebellar pathology, and occasional atypical findings may mimic other neurologic conditions, necessitating careful interpretation in the clinical context.

Among the 40 patients, anti-Yo antibodies were confirmed in serum only in 14 cases, CSF only in 2 cases, and in both serum and CSF in 20 cases, while 4 cases lacked documentation regarding the source of antibody detection. This highlights the diagnostic utility of testing both serum and CSF to increase the likelihood of detecting anti-Yo antibodies in suspected PCD.

Management generally combined definitive tumor therapy (often debulking surgery with platinum/taxane chemotherapy) and immunomodulatory treatments (high-dose steroids, IVIG, PLEX). Although most patients stabilized rather than experienced full neurologic recovery, a subset—8 of 38 cases (21%)—demonstrated significant neurologic improvement. For example, one patient regained independent ambulation at one year (Abdulaziz et al. 2018), another walked independently by two months with only minimal residual ataxia (Bhargava et al. 2014), and several others restored gait or showed marked improvement in coordination and balance (İlhan et al. 2021; Tanaka et al. 1992; Hu et al. 2020; Mirallas et al. 2020; Yan et al. 2018; Yang et al. 2025). These findings suggest that approximately 1 in 5 anti-Yo PCD patients may achieve functional recovery, notably higher than the < 10% recovery rate previously reported (Bradley et al. 2008).

Analyzing the eight cases with significant neurological recovery reveals a pattern: patients who improved tended to receive early intervention, targeting both the underlying malignancy and the immune process within a narrow window (ideally within 1–3 months) of symptom onset. Most had normal baseline MRIs (i.e., no cerebellar atrophy) and were treated with multimodal therapy, including surgery, chemotherapy, immunosuppressants (e.g., IVIG, steroids), and often structured rehabilitation. For example, in the case by Yang et al. (2025) a 62-year-old patient with lung cancer received prednisone and IVIG within 1–2 months of symptom onset, followed promptly by chemoradiation—resulting in resolution of her nystagmus and normalization of gait.

In contrast, patients who remained disabled generally experienced delays in diagnosis or incomplete treatment beyond this critical window, often accompanied by evidence of neuronal loss on imaging. For instance, Ćerimagić and Džananović (2020) describe a 61-year-old woman whose cerebellar symptoms persisted for approximately 10 months before receiving any tumor-directed or immunosuppressive therapy. Despite aggressive treatment with plasmapheresis, cyclophosphamide, azathioprine, and IVIG, she remained wheelchair-bound with severe ataxia and dysarthria.

Together, these examples suggest a clear pattern: once ataxia persists beyond approximately three to six months without definitive cancer-directed therapy and immunomodulation, irreversible Purkinje cell loss often ensues, and subsequent interventions fail to restore neurologic function. Clinicians should therefore recognize that initiating early, combined oncologic and PCD-specific therapy is crucial to maximize the chance of recovery.

Beyond conventional treatments such as tumor resection, chemotherapy, plasma exchange (PLEX), IVIG, steroids, and other immunosuppressive strategies, whole brain radiation therapy (WBRT) has been reported in a unique anti-Yo PCD case by Westerlinck et al. (2022). The patient—a 31-year-old woman with breast cancer-associated PCD—developed severe neurologic deficits, including expressive aphasia, paraparesis, and cerebellar tremor, despite tumor regression and standard immunosuppressive therapy. Concerned about irreversible damage, the clinical team proceeded with mastectomy followed by WBRT (10 Gy in 5 fractions), not for metastatic disease but rather to suppress the cerebellar autoimmune response. Following this combined approach, her neurologic decline stabilized, and she experienced gradual recovery of speech and motor function over the subsequent months (Westerlinck et al. 2022). This exceptional case raises the possibility that cranial irradiation may exert immune-modulating or neuroprotective effects in rapidly progressive anti-Yo PCD. While promising, the potential role of WBRT in this context remains speculative and warrants further clinical investigation.

Our patient’s presentation was typical in several ways: a middle-aged woman with subacute onset of vertigo, diplopia, and gait instability evolving over three months, accompanied by initially unremarkable imaging and a positive anti-Yo antibody result that prompted a thorough malignancy workup. Her treatment timeline and clinical course align with the patterns observed in prior cases. She first developed cerebellar symptoms preceding her initial presentation, but targeted immunotherapy—including five sessions of plasma exchange, IVIG, and high-dose steroids—was not initiated until approximately three months after symptom onset, when high-titer anti-Yo antibodies were confirmed. Definitive tumor-directed therapy, consisting of hysterectomy, bilateral salpingo-oophorectomy, and pelvic lymphadenectomy followed by adjuvant carboplatin/paclitaxel, was further delayed until nearly seven months after initial neurologic symptoms emerged.

This delay beyond the critical 3-to-6-month window—associated with meaningful recovery in other anti-Yo PCD cases—likely contributed to her outcome: modest improvements in speech and coordination, but persistent non-ambulatory status. Her course reinforces the emerging observation that delays of six months or more often coincide with irreversible cerebellar injury, underscoring the importance of early recognition and dual-targeted treatment.

Our case is unique among reported anti-Yo PCD cases in that it is the first to document truly isolated nodal malignancy of presumed gynecologic origin confirmed through comprehensive surgical exploration, with no identifiable primary tumor. In Abdulaziz et al. (2018) a 68-year-old woman underwent axillary lymph node biopsy revealing metastatic carcinoma, and subsequent bilateral breast biopsies were negative; however, no pelvic or gynecologic exploration was performed to rule out an alternative primary source. Similarly, Chatham and Niravath (2021) reported a 61-year-old woman with isolated axillary node metastases who underwent mastectomy without detection of a breast tumor, and no further evaluation for a gynecologic origin was pursued. In the case by Elomrani et al. (2014) an 80-year-old woman underwent biopsy of an inguinal lymph node that revealed poorly differentiated adenocarcinoma with a gynecologic immunophenotype, yet she did not undergo hysterectomy or salpingo-oophorectomy, leaving the possibility of an occult ovarian primary unresolved. By contrast, our patient underwent robotic hysterectomy, bilateral salpingo-oophorectomy, and pelvic lymphadenectomy, with histopathology confirming clear cell carcinoma isolated to two common iliac lymph nodes and no malignancy found in the uterus, ovaries, or fallopian tubes. To the best of our knowledge, this represents the first anti-Yo PCD case with definitive exclusion of a gynecologic primary through complete surgical staging, supporting a diagnosis of isolated nodal metastases of presumed gynecologic origin.

The diagnostic challenge in such situations is profound. Even with serum and CSF anti-Yo positivity—strongly suggestive of breast or gynecologic malignancy—tumors may remain occult on routine imaging. In our patient, contrast CT and endometrial biopsy were unrevealing. Only FDG-PET/CT detected hypermetabolic iliac nodes. This highlights the value of FDG-PET/CT when standard imaging fails to localize a primary lesion in suspected PCD. If PET identifies isolated lymphadenopathy, empiric surgical exploration (e.g., lymphadenectomy plus removal of potential sites of origin) may be necessary to confirm the diagnosis and guide treatment. To the best of our knowledge, only two patients—Hu et al. (2020) and Dou et al. (2024)—with anti-Yo-positive PCD remained tumor-negative after exhaustive imaging and biopsy workups. Even in an exceptional report by Hasadsri et al. (2013), a 49-year-old man with anti-Yo PCD underwent extensive evaluation (including CT, MRI, and PET), yet no malignancy was identified ante mortem; only at autopsy was a 0.8 cm large-cell lung adenocarcinoma discovered, confirming an occult tumor hidden from clinical detection. Such “tumor-negative” cases therefore likely represent occult malignancies that either spontaneously regressed or remained below detection thresholds rather than true, permanent absence of cancer.

Our report expands the recognized spectrum of gynecologic malignancies associated with anti-Yo PCD by documenting a nodal clear cell carcinoma without an identifiable primary. When anti-Yo antibodies are detected but imaging is negative, clinicians should consider advanced metabolic imaging (FDG-PET/CT) and maintain a low threshold for surgical exploration of potential primary sites—even in the absence of radiographic or endometrial abnormalities. Early tumor localization and removal may limit irreversible Purkinje cell loss, although the overall prognosis for neurologic recovery remains guarded.

## Conclusion

In conclusion, this case highlights the importance of maintaining a high index of suspicion for breast or gynecologic malignancies in patients presenting with subacute cerebellar dysfunction and anti-Yo antibodies, even when initial imaging studies are unrevealing. Advanced metabolic imaging such as FDG-PET/CT can uncover occult nodal disease that eludes conventional CT or MRI, and when PET identifies isolated lymphadenopathy without a clear primary, empiric surgical exploration—including hysterectomy, bilateral salpingo-oophorectomy, and lymphadenectomy—may be necessary to confirm the diagnosis. Although multimodal therapy (surgical resection, chemotherapy, plasma exchange, IVIG, and corticosteroids) can stabilize neurologic decline, irreversible Purkinje cell injury often leaves patients with significant residual deficits; therefore, early tumor localization and prompt treatment are crucial to optimizing neurologic outcomes, despite the overall guarded prognosis for full cerebellar recovery.

## Data Availability

No datasets were generated or analysed during the current study.
